# Bayesian Estimation-Based Pedestrian Tracking in Microcells

**DOI:** 10.1155/2013/187479

**Published:** 2013-09-24

**Authors:** Yoshiaki Taniguchi, Masahiro Sasabe, Satoshi Aihara, Hirotaka Nakano

**Affiliations:** ^1^The Cybermedia Center, Osaka University, Toyonaka 560-0043, Japan; ^2^Graduate School of Engineering, Osaka University, Suita 565-0871, Japan; ^3^Graduate School of Information Science and Technology, Osaka University, Suita 565-0871, Japan

## Abstract

We consider a pedestrian tracking system where sensor nodes are placed only at specific points so that the monitoring region is divided into multiple smaller regions referred to as microcells. In the proposed pedestrian tracking system, sensor nodes composed of pairs of binary sensors can detect pedestrian arrival and departure events. In this paper, we focus on pedestrian tracking in microcells. First, we investigate actual pedestrian trajectories in a microcell on the basis of observations using video sequences, after which we prepare a pedestrian mobility model. Next, we propose a method for pedestrian tracking in microcells based on the developed pedestrian mobility model. In the proposed method, we extend the Bayesian estimation to account for time-series information to estimate the correspondence between pedestrian arrival and departure events. Through simulations, we show that the tracking success ratio of the proposed method is increased by 35.8% compared to a combinatorial optimization-based tracking method.

## 1. Introduction

Wireless sensor network technology has attracted considerable attention in recent years. Pedestrian tracking is one of the most promising applications of wireless sensor networks. For example, the ability to evaluate the trajectories of pedestrians in shopping malls or event sites would allow vendors and event organizers to arrange goods or exhibitions more effectively so that they attract the attention of more people. A variety of sensors have been considered for implementation of pedestrian tracking by using wireless sensor networks, such as cameras [1, 2], laser range scanners [3], and binary sensors [4–12].

Binary sensors, such as infrared and pressure sensors, are among the most simple, inexpensive, and energy-efficient sensors. Even though they can detect only the presence or absence of pedestrians within the sensing range, they have found widespread adoption for various applications. A number of pedestrian tracking methods using a binary sensor network have been proposed [4–12]. However, the above-mentioned studies have relied on the assumption that sensor nodes are deployed uniformly so that the sensing region covers the entire monitoring region, which requires a large number of sensor nodes.

**Figure 1:**
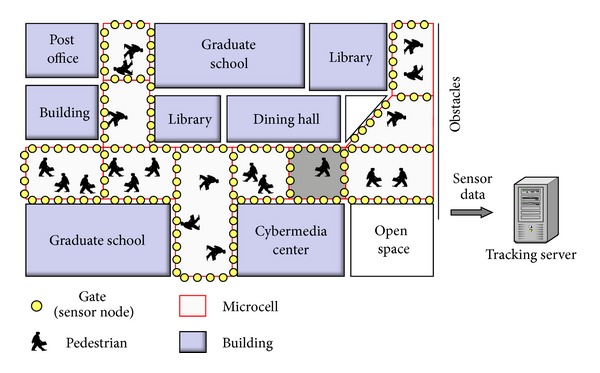
Pedestrian tracking system.

In this paper, we consider a pedestrian tracking system where sensor nodes are placed only at specific points in the monitoring region, so that the monitoring region is divided into multiple smaller regions referred to as *microcells*. Each sensor node is composed of a pair of binary sensors coupled with a wireless communication device, and its function is to detect pedestrian arrival and departure events [13–15]. We refer to the locations at which sensor nodes are placed as *gates*.  Figure 1 shows a schematic representation of the proposed tracking system implemented in a university campus. In the tracking system, sensor information is collected by a tracking server through a wireless network, and the tracking server estimates pedestrian trajectories based on sensor information.

In this paper, we focus on pedestrian tracking in a single microcell. First, we investigate actual pedestrian trajectories in a microcell based on video sequences, after which we develop a pedestrian mobility model. Next, we show that pedestrians move approximately in a straight line and that pedestrian velocities in a microcell follow a normal distribution. These results suggest that we can estimate pedestrian trajectories in a microcell by observing pedestrian arrival and departure events at specific points (i.e., gates) and estimating the correspondence between these events. Next, we propose a novel pedestrian tracking method based on the developed pedestrian mobility model. In the proposed method, we extend the Bayesian estimation to account for time-series information to estimate the correspondence between pedestrian arrival and departure events. We evaluate the performance of proposed method in a variety of situations by using actual and artificial trajectories through simulation experiments.

The rest of this paper is organized as follows. In Section 2, we present the observation results of actual pedestrian trajectories and develop a pedestrian mobility model in a microcell. In Section 3, we propose a Bayesian estimation-based pedestrian tracking method. We evaluate the performance of proposed method in Section 4. Finally, we conclude this paper with possible directions of future research in Section 5.

## 2. Modeling Pedestrian Mobility in a Microcell

In this section, we investigate actual pedestrian trajectories in a microcell by analyzing video sequences and develop a pedestrian mobility model.

### 2.1. Observation Overview

**Figure 2:**
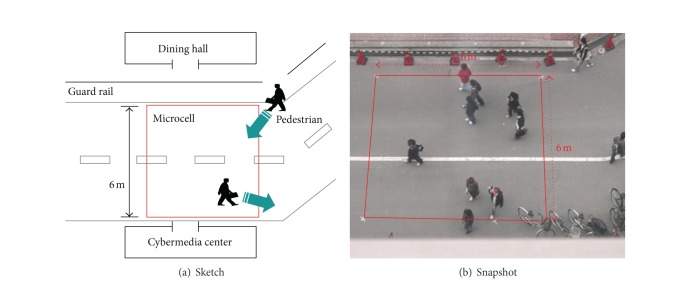
Observation environment.

We took a video of actual pedestrians walking on a road to investigate actual pedestrian trajectories in a microcell. The video was taken from the 7th floor of the building of the Cybermedia Center, Osaka University, Japan.  Figure 2 shows a sketch and a snapshot of the observation environment, which corresponds to the gray microcell and its surrounding environment in Figure 1. In the observation environment, there are a road and two buildings on both sides: the Cybermedia Center at the near end, and the dining hall is at the opposite side. To go to the dining hall from the road, pedestrians have to move to avoid the guard rails. The duration of the recorded video was 600 s, and the sampling rate was 500 ms. In the video sequences, a 6 × 6 m square region was chosen as a microcell (indicated by the quadrangular region enclosed by red lines in Figure 2). For the duration of the video, 125 pedestrians passed through the microcell. Therefore, the overall arrival rate was 125/600 = 0.208 pedestrians/s. Below, we refer to this rate as *cell arrival rate* and denote it as *λ*. We manually obtained the locations of all pedestrians in all frames. Then, we obtained the trajectory of each pedestrian by interlinking the pedestrians' locations in ascending order in the observation period. To convert pedestrian trajectories from the camera coordinate system to a world coordinate system in overhead view, we used the projective transformation method [16]. We also obtained pedestrian velocities between two successive locations for each pedestrian.

### 2.2. Pedestrian Trajectories in a Microcell

**Figure 3:**
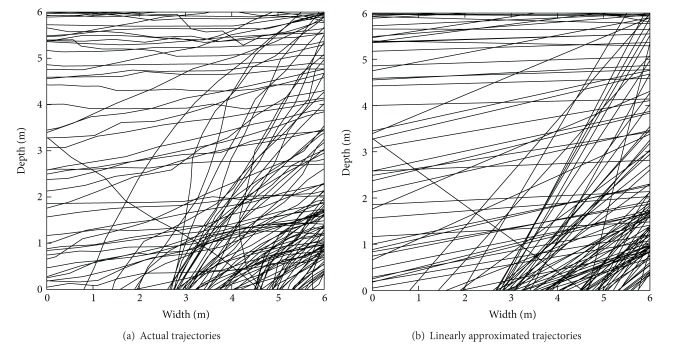
Actual and approximated pedestrian trajectories.

**Figure 4:**
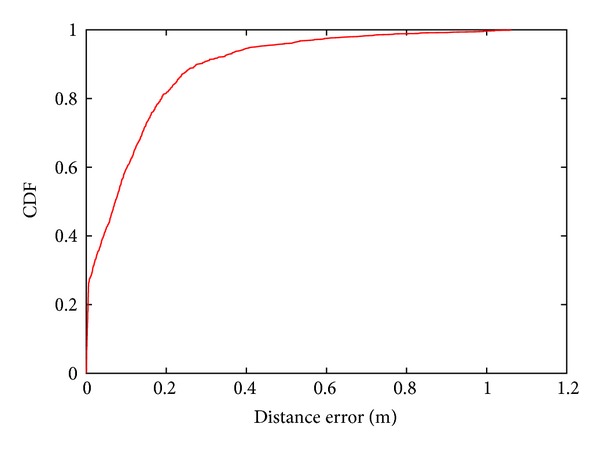
Cumulative distribution function of the distance error between the actual locations and the corresponding approximated locations.

Figure 3(a) shows the actual pedestrian trajectories, while Figure 3(b) shows linearly approximated trajectories drawn from the arrival location of each pedestrian to the corresponding departure location.  Figure 4 shows the cumulative distribution function of the distance error obtained by comparing the actual locations with the corresponding approximated locations in the sampling interval. As shown in Figure 4, the distance error is within 0.30 m in 90% of the cases. Since 0.30 m is a relatively small error compared to the size of pedestrians, the linear approximation is considered sufficient for obtaining pedestrian trajectories in a microcell. These results indicate that we can estimate pedestrian trajectories by obtaining only the pedestrian arrival and departure locations.

### 2.3. Pedestrian Velocities in a Microcell

**Figure 5:**
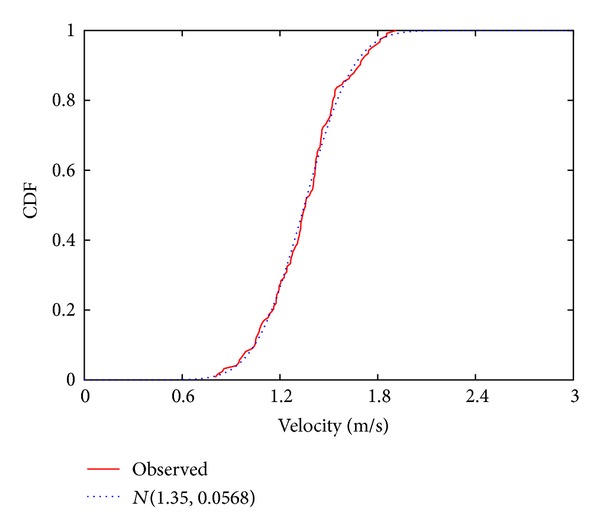
Cumulative distribution function of pedestrian velocities and its approximation with a normal distribution.

Figure 5 shows the cumulative distribution function of the pedestrian velocities obtained in the sampling interval. From the observation results, the mean pedestrian velocity $\bar{v}$ and the variance *σ* ^2^ were calculated to be 1.35 m/s and 5.68 × 10^−2^ m^2^/s^2^, respectively. In the same figure, we also show a normal distribution $N(\bar{v},\sigma^{2})$ [17] which approximates the observation results. The experimentally observed pedestrian velocities are found to be well approximated by the normal distribution. There are also other distributions which share similar trends with the observed cumulative distribution function, such as the log-normal distribution [18], the gamma distribution [19], and the Cauchy distribution [20]. We fitted these distributions to the observed cumulative distribution function and calculated the mean square error for each case, in which the normal distribution yielded the smallest error. Therefore, we considered that pedestrian velocities in a microcell follow a normal distribution.

We use the normal distribution to model pedestrian velocities. Therefore, the probability density function of pedestrian velocities is given by

(1) $$\begin{array}{c} p_{\text{vel}}\left(v\right)=\frac{1}{\sqrt{2\pi }\sigma }\exp\left(-\frac{{\left(v-\bar{v}\right)}^{2}}{2\sigma^{2}}\right), \end{array}$$

where *v* is pedestrian velocity. Therefore, the probability density function of the pedestrian transit time required for a pedestrian to cover a distance *D* can be written as follows:

(2) $$\begin{array}{c} p_{\text{time}}\left(\tau ,D\right)=\frac{D}{\tau^{2}}\frac{1}{\sqrt{2\pi }\sigma }\exp\left(-\frac{{\left(\left(D/\tau \right)-\bar{v}\right)}^{2}}{2\sigma^{2}}\right), \end{array}$$

where *τ* is the pedestrian transit time. The details of the derivation of (2) from (1) are presented in the appendix.

### 2.4. Pedestrian Gate Transition and Gate Arrival Probabilities

**Figure 6:**
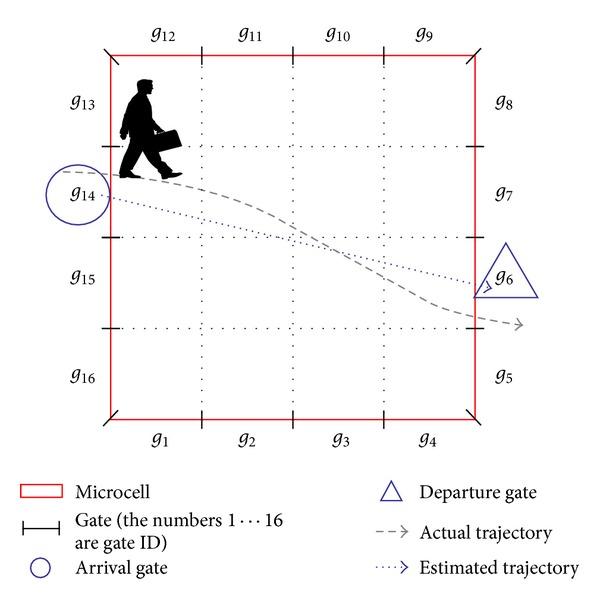
Relationship between a microcell and gates (*n* = 16).

To investigate the relationships between the pedestrian arrival and departure locations, we divide the microcell into equal-sized grids and obtain *n* segments (gates) at the boundary. Below, the *i*th gate is denoted as *g*_*i*_, and the set of gates in the microcell is denoted as *𝒢*.  Figure 6 shows the relationship between microcell and gates for *n* = 16. If a pedestrian passes through the microcell as shown in Figure 6, the arrival and departure events are detected at gates *g*_14_ and *g*_6_, respectively.

We refer to the probability that a pedestrian arrives at gate *g*_*a*_ and departs from gate *g*_*d*_ as *gate transition probability* and denote it by *p*_transit_(*g*_*a*_, *g*_*d*_). The gate transition probability is calculated from observation as follows:

(3) $$\begin{array}{c} p_{\text{transit}}\left(g_{a},g_{d}\right)=\frac{m\left(g_{a},g_{d}\right)}{m_{\text{all}}}, \end{array}$$

where *m*(*g*_*a*_, *g*_*d*_) indicates the number of pedestrians who arrive at gate *g*_*a*_ and depart from gate *g*_*d*_ and *m*_all_ indicates the total number of pedestrians in the video sequences. The set of gate transition probabilities is denoted by *𝒫*_transit_ = {*p*_transit_(*g*_*a*_, *g*_*d*_) | *g*_*a*_ ∈ *𝒢*,  *g*_*d*_ ∈ *𝒢*}. In addition, we refer to the probability that a pedestrian arrives at gate *g*_*a*_ as *gate arrival probability* and denote it by *p*_arr_(*g*_*a*_). The gate arrival probability is calculated from observation as follows:

(4) $$\begin{array}{c} p_{\text{arr}}\left(g_{a}\right)=\frac{m\left(g_{a}\right)}{m_{\text{all}}}, \end{array}$$

where *m*(*g*_*a*_) indicates the number of pedestrians who arrive at gate *g*_*a*_. The set of gate arrival probabilities is denoted by *𝒫*_arr_ = {*p*_arr_(*g*_*a*_) | *g*_*a*_ ∈ *𝒢*}.

**Figure 7:**
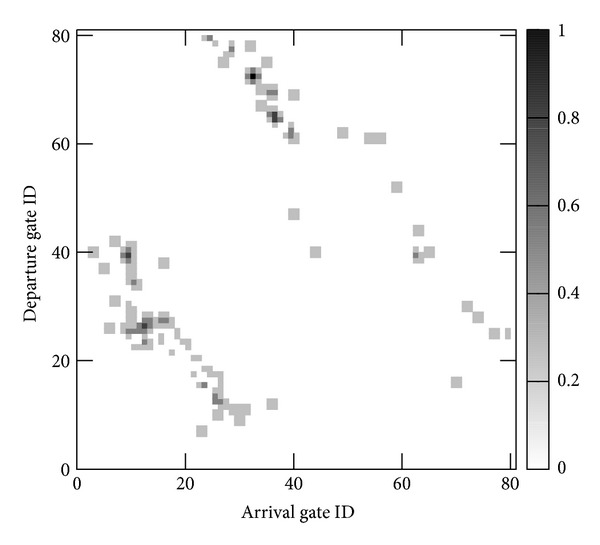
Distribution of gate transition probabilities *𝒫*_transit_ (*n* = 80).

**Figure 8:**
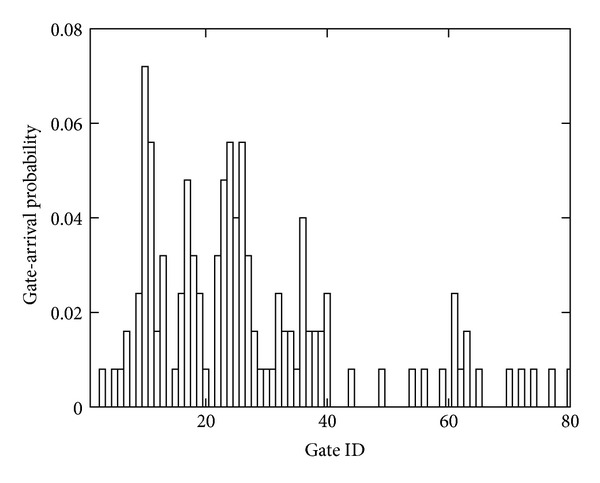
Distribution of gate arrival probabilities *𝒫*_arr_ (*n* = 80).

Figures 7 and 8, respectively, show the distributions of gate transition and gate arrival probabilities when *n* is set to 80, that is, when the width of each gate is 0.3 m. The gate transition probabilities are symmetrical with respect to the line *y* = *x*, and most gate transition probabilities are zero. These results indicate that there are few bidirectional paths which are frequently used and that the gate transition probabilities do not follow a uniform distribution in real-world environments. From Figure 8, we can see that the distribution of gate arrival probabilities is also nonuniform. The gate arrival probability is zero at 40% of the gates and takes a high value at some adjacent gates.  Table 1 summarizes the parameters and their values in the observed environment.

### 2.5. Summary of Pedestrian Mobility in a Microcell

Through these experiments we found that (1) pedestrians move approximately along straight lines, (2) pedestrian velocities follow a normal distribution, and (3) gate transition and gate arrival probabilities do not follow a normal distribution. We conjecture that (1) stems from the fact that pedestrians aim to reach their destination along the shortest path. Furthermore, we assume that (2) is a general characteristic of pedestrian movement. Finally, (3) appears to depend on the environment, for example, the locations and configuration of facilities. Although our experiment provides only a single example, we expect that this new knowledge will be applicable in other similar environments.

## 3. Proposed Pedestrian Tracking Method

Considering the model presented in the previous section, in this section we propose a Bayesian estimation-based pedestrian tracking method.

### 3.1. Overview

First, we present an overview of the tracking system. In this paper, we focus on pedestrian tracking in a microcell constituting a small part of the entire monitoring region (Figure 1). We should note here that the microcell is assumed to be sufficiently small area so that pedestrian trajectories can be approximated linearly. The border of the microcell is divided into *n* segments (gates), and a sensor node is placed at each gate. Each sensor node is composed of a pair of binary sensors with a wireless communication device, and its function is to detect pedestrian arrival and departure events. Here, note that we consider that an arrival/departure event is detected at the location of the sensor node. We denote an arrival event detected by the sensor node at gate *g*_*a*_ at time *t*_*i*_ by *e*_arr_(*g*_*a*_, *t*_*i*_) and a departure event detected by the sensor node at gate *g*_*d*_ at time *t*_*j*_ by *e*_dep_(*g*_*d*_, *t*_*j*_). Sensor information about arrival/departure events and the timing of events is collected by a tracking server, which estimates the pedestrian trajectories based on this information.

Since each arrival and departure event is observed independently at each gate, matching arrival and departure events are required. In this study, matching is performed when the tracking server obtains information on a departure event. When such an event is detected, the tracking server must have more than one arrival event as a candidate for matching the departure event. To select the appropriate arrival event from a set of candidate arrival events, we propose a matching method based on the Bayesian estimation [21] which is extended to account for time-series information.

### 3.2. Matching Likelihood

Before presenting the proposed tracking method, we first derive the likelihood that departure event *e*_dep_(*g*_*d*_, *t*_*j*_) corresponds to arrival event *e*_arr_(*g*_*a*_, *t*_*i*_). We refer to this as *matching likelihood* and denote it by *p*(*e*_arr_(*g*_*a*_, *t*_*i*_) | *e*_dep_(*g*_*d*_, *t*_*j*_)). We can calculate the matching likelihood by using the following theorem.

**Theorem 1**

When the tracking system is in a steady state, the matching likelihood *p*(*e*_*arr*_(*g*_*a*_, *t*_*i*_) | *e*_*dep*_(*g*_*d*_, *t*_*j*_)) is written as follows:

(5) p(earr(ga,ti) ∣ edep(gd,tj))  =ptime(tj−ti,d(ga,gd))ptransit(ga,gd),

where *d*(*g*_*a*_, *g*_*d*_) is the distance between gates *g*_*a*_ and *g*_*d*_.

**Proof**

Let *e*_arr_(*g*_*a*_) be an arrival event detected at gate *g*_*a*_ and *e*_dep_(*g*_*d*_) a departure event detected at gate *g*_*d*_. According to the Bayes theorem, we can obtain the relationship between the conditional and marginal probabilities of stochastic events *e*_arr_(*g*_*a*_) and *e*_dep_(*g*_*d*_) as

(6) p(earr(ga) ∣ edep(gd))  =p(edep(gd) ∣ earr(ga))p(earr(ga))p(edep(gd)).

By taking time into account, (6) can be extended as follows:

(7) p(earr(ga,ti) ∣ edep(gd,tj))  =p(edep(gd,tj) ∣ earr(ga,ti))p(earr(ga,ti))p(edep(gd,tj)).

In a steady state, we assume that the occurrence rate of arrival and departure events is independent of time. As a result, (7) becomes

(8) p(earr(ga,ti) ∣ edep(gd,tj)) =p(edep(gd,tj−ti) ∣ earr(ga))p(earr(ga))p(edep(gd)).

Let *v* be the velocity of a pedestrian passing between two gates. Also *p*(*e*_dep_(*g*_*d*_, *t*_*j*_ − *t*_*i*_) | *e*_arr_(*g*_*a*_)) in (8) can be denoted by the product of the probability distribution *p*(*d*(*g*_*a*_, *g*_*d*_)/*v*) of the transit time between gates *g*_*a*_ and *g*_*d*_ and the probability of pedestrian transit from gate *g*_*a*_ to gate *g*_*d*_ in the steady state. Consequently, (8) can be rewritten as follows by using (6):

(9) p(earr(ga,ti) ∣ edep(gd,tj)) =p(d(ga,gd)v)p(edep(gd) ∣ earr(ga))p(earr(ga))p(edep(gd)) =ptime(tj−ti,d(ga,gd))p(earr(ga) ∣ edep(gd)).

In (9), *p*(*e*_arr_(*g*_*a*_) | *e*_dep_(*g*_*d*_)) denotes the probability that a pedestrian moves from *g*_*a*_ to *g*_*d*_ in the steady state, that is, gate transition probability *p*_transit_(*g*_*a*_, *g*_*d*_). Therefore, we obtain the matching likelihood as in (5).

### 3.3. Bayesian Estimation-Based Tracking Method

We now propose a tracking method using the results described in the previous section. We assume that the distribution of gate transition probabilities *𝒫*_transit_, mean velocity $\bar{v}$, and variance of velocity *σ* ^2^ is estimated a priori by pre-learning.

The tracking server maintains a set of candidate arrival events *ℰ*_arr_. When the tracking server obtains information about an arrival event, it adds the arrival event to the set of candidate arrival events *ℰ*_arr_ for future matching. Conversely, when the tracking server obtains information about a departure event *e*_dep_(*g*_*d*_, *t*_*j*_), it starts matching the departure event to arrival events in the set of candidate arrival events *ℰ*_arr_. Here, we denote the *k*th candidate arrival event in the set by *e*_arr_^(*k*)^(*g*, *t*) ∈ *ℰ*_arr_. The tracking server first calculates the matching likelihood *p*(*e*_arr_^(*k*)^(*g*, *t*) | *e*_dep_(*g*_*d*_, *t*_*j*_)) for each candidate arrival event *e*_arr_^(*k*)^(*g*, *t*) ∈ *ℰ*_arr_ using (5). Next, it selects the candidate arrival event *e*_arr_^(*k*_max⁡_)^(*g*_*a*_max⁡__, *t*_*i*_max⁡__) with the highest matching likelihood as the arrival event corresponding to departure event *e*_dep_(*g*_*d*_, *t*_*j*_):

(10) earr(kmax⁡)(gamax⁡,timax⁡) =arg max⁡earr(k)(g,t)∈ℰarrp(earr(k)(g,t) ∣ edep(gd,tj)).

After matching, the tracking server estimates a line from the location of gate *g*_*a*_max⁡__ to the location of gate *g*_*j*_ as the pedestrian trajectory.

Since there is a limitation on the transit time in a microcell, *ℰ*_arr_ on the tracking server does not need to contain all arrival events. In the proposed method, candidate arrival events which have occurred prior to a certain period of time are removed from *ℰ*_arr_. In addition, the candidate arrival event *e*_arr_^(*k*_max⁡_)^(*g*_*a*_max⁡__, *t*_*i*_max⁡__) is removed from *ℰ*_arr_ after matching depending on its *matching reliability r*, which is defined as follows:

(11) $$\begin{array}{c} r=\frac{p\left(e_{\text{arr}}^{\left(k_{\max}\right)}\left(g_{a_{\max}},t_{i_{\max}}\right)\;|\;e_{\text{dep}}\left(g_{d},t_{j}\right)\right)}{\sum_{e_{\text{arr}}^{\left(k\right)}(g,t)\in {ℰ}_{\text{arr}}}p\left(e_{\text{arr}}^{\left(k\right)}\left(g,t\right)\;|\;e_{\text{dep}}\left(g_{d},t_{j}\right)\right)}. \end{array}$$

After matching, if the matching reliability is higher than a predefined reliability threshold, that is, *r* ≥ *r*_th_, candidate arrival event *e*_arr_^(*k*_max⁡_)^(*g*_*a*_max⁡__, *t*_*i*_max⁡__) is removed from *ℰ*_arr_.

**Figure 9:**
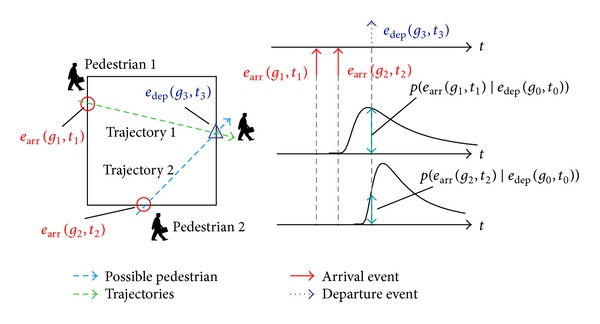
Example of pedestrian tracking in a microcell.

Figure 9 shows an example of pedestrian tracking in a microcell. In this example, a pedestrian arrives at the microcell at gate *g*_1_ at time *t*_1_, after which another pedestrian arrives at gate *g*_2_ at time *t*_2_. Therefore, the tracking server maintains a set of two candidate arrival events: *ℰ*_arr_ = {*e*_arr_^(1)^(*g*_1_, *t*_1_), *e*_arr_^(2)^(*g*_2_, *t*_2_)}. After a certain amount of time, a pedestrian departs from the microcell at gate *g*_3_ at time *t*_3_. At this stage, there are two possible pedestrian trajectories to gate *g*_3_: trajectory 1 from gate *g*_1_ or trajectory 2 from gate *g*_2_. Among these candidate trajectories, the matching likelihood for the trajectory from gate *g*_1_ is higher than that from gate *g*_2_. Therefore, trajectory 1 is selected as the tracking result. If the matching reliability *r* is higher than the reliability threshold, candidate arrival event *e*_arr_^(1)^(*g*_1_, *t*_1_) is removed from *ℰ*_arr_.

## 4. Performance Evaluation

In this section, we evaluate the proposed method. Although our method utilizes linearly approximated pedestrian trajectories, which are slightly different from actual pedestrian trajectories, the difference is negligible, as shown in Section 2. Therefore, in this section we focus on the estimation accuracy of the correspondence between arrival and departure events to evaluate the performance of the proposed method. We define *tracking success ratio* as the ratio of the number of successful matches to the total number of matches and use this ratio as a performance evaluation index.

### 4.1. Comparison with a Combinatorial Optimization-Based Method Using Actual Pedestrian Trajectories

First, we evaluate the proposed method using actual pedestrian trajectories obtained in Section 2. To evaluate the basic performance of the proposed method, we use the parameters and their values listed in Table 1. The results are compared with those obtained using a combinatorial optimization-based method [22] (referred to as the compared method below), which uses the mean pedestrian velocity $\bar{v}$ for matching arrival and departure events. In the compared method, the objective function *f* is written as

(12) $$\begin{array}{c} f=\sum_{e_{\text{arr}}(g_{a},t_{i}),e_{\text{dep}}(g_{d},t_{j})\in ℰ}\left|\left(t_{j}-t_{i}\right)-\frac{d\left(g_{d},g_{a}\right)}{\bar{v}}\right|, \end{array}$$

where *ℰ* is a set of arrival and departure events in a specific time period. The combination of pairs of arrival and departure events for which the objective function *f* is minimized is selected as the matching result. Although the accuracy of matching increases if the distribution of velocities is taken into account, the compared method has a larger search space and higher complexity.

We calculate the tracking success ratio for both the proposed method and the compared method using the data set of actual pedestrian trajectories. The tracking success ratio of the proposed method is 1, while that of the compared method reaches a maximum of 0.736, amounting to an improvement of 35.8% in tracking success ratio with the proposed method. It should, however, be noted that the tracking success ratio in this evaluation reaches 1 since the cell arrival rate in the actual data set is relatively low, and we assume that the parameters of the proposed method are known. In the following sections, we evaluate the proposed method in a variety of situations.

### 4.2. Effect of Reliability Threshold

In this section, we present a simulation using artificial pedestrian trajectories to investigate the performance of the proposed method in a variety of situations. In the simulation, the size of the microcell is set to 6 × 6 m and the number of gates *n* is set to 80. A pedestrian arrives at gate *g*_*k*_ in accordance with a Poisson process with a gate arrival rate *λ*_*k*_. The gate arrival rate *λ*_*k*_ is the product of the cell arrival rate *λ* and gate arrival probability *p*_arr_(*g*_*k*_) ∈ *𝒫*_arr_. The departure gate of the pedestrian is determined according to gate transition probabilities *𝒫*_transit_, where we assume that the pedestrian moves to the departure gate along a straight line. The pedestrian velocities follow a normal distribution. In this simulation, we use the parameter settings shown in Table 1 for the mean velocity $\bar{v}$, variance of velocity *σ* ^2^, gate transition probabilities *𝒫*_transit_, and gate-arrival probability *𝒫*_arr_. The observation duration is set to 600 s, and the cell arrival rate *λ* is set to 1, 2, or 3 pedestrians/s. The correspondence between arrival and departure events is estimated using the proposed method. The following results are averaged over 100 iterations.

**Figure 10:**
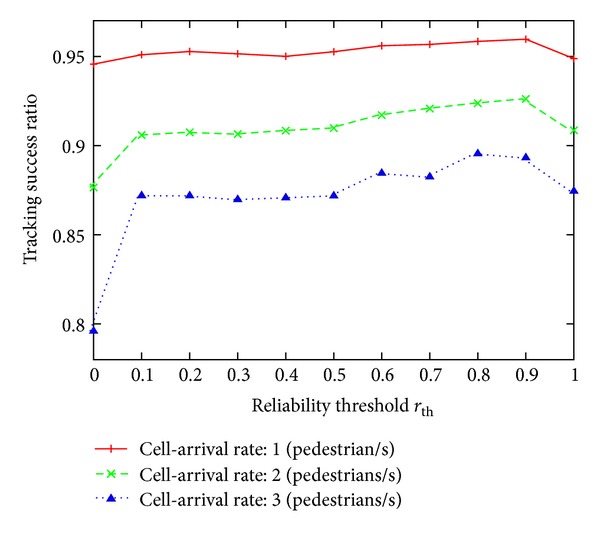
Tracking success ratio plotted against the reliability threshold *r*_th_.

Figure 10 shows a plot of the relation between tracking success ratio and the reliability threshold *r*_th_, where we can see that there is an optimal value for the reliability threshold which maximizes the tracking success ratio. The optimal reliability threshold was between 0.8 and 0.9 in this evaluation. The reason why there is an optimal value is as follows. When the reliability threshold *r*_th_ is too low, candidate arrival events are easily deleted from the set of candidate arrival events even in the case of a mismatch. As a result, the number of mismatches increases and the tracking success ratio decreases. In contrast, when the reliability threshold is too high, more candidate arrival events remain in the set of candidate arrival events, increasing the possibility for a mismatch.

In addition, as shown in Figure 10, the tracking success ratio is higher for lower cell arrival rates. This is because the number of candidate arrival events for matching a departure event is higher for higher cell arrival rates, and as a result it becomes difficult to estimate the correct arrival event.

### 4.3. Effect of Distribution of Gate Transition Probabilities

Then, we investigate how the distribution of gate transition probabilities *𝒫*_transit_ affects the tracking success ratio. We used two types of gate transition probability distributions: a uniform distribution and an observation-based distribution (Figure 7). In this section, the reliability threshold *r*_th_ is set to 0.9.

**Figure 11:**
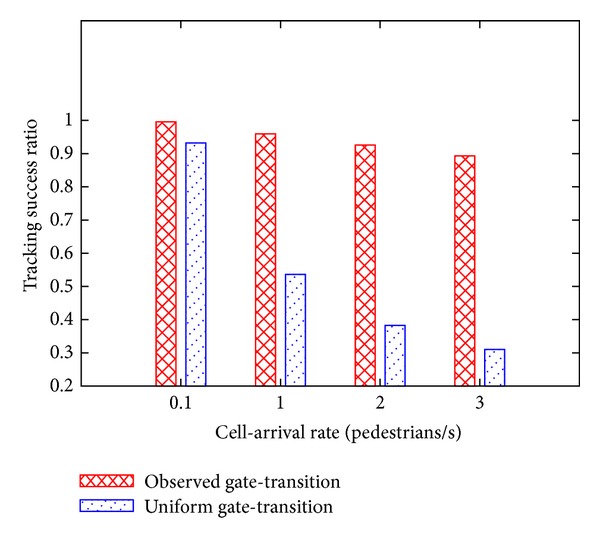
Tracking success ratio plotted against the distribution of gate transition probabilities.

Figure 11 shows the relationship between the tracking success ratio, the pedestrian cell arrival rate, and the distribution of gate transition probabilities. The tracking success ratio is lower for higher cell arrival rates, and this trend is independent of the distribution of transition probabilities. However, when we used the observed distribution of gate transition probabilities, the tracking success ratio remains much higher for different cell arrival rates as compared to the results obtained with the uniform distribution of gate transition probabilities. From this result, we conclude that the proposed method is more suitable for situations where pedestrian transition is nonuniform.

### 4.4. Effect of Learning Period

In the previous sections, we used the same values for the parameters in the mobility model in the simulation and for the parameters in the proposed method. In practical situations, the parameters of the proposed method are obtained by prelearning. In this section, we investigate the effect of the duration of the learning period on the tracking success ratio of the proposed method. In this simulation, the observation period is set to 3600 s and the cell arrival rate is set to 2 pedestrians/s. We calculate the distribution of gate transit probabilities *𝒫*_transit_ using the beginning (i.e., the learning period) of the observation period and then evaluate the tracking success ratio of the entire observation period.

**Figure 12:**
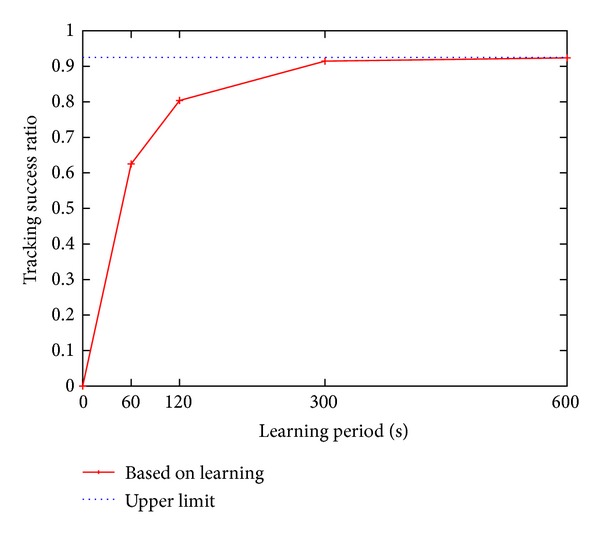
Tracking success ratio during a 3600 s simulation period plotted against the learning period.

Figure 12 shows the tracking success ratio plotted against the learning period. To show that there is an upper limit to the tracking success ratio, we also show the tracking success ratio when the entire observed period of 3600 s is used as a learning period (Figure 12). The tracking success ratio clearly increases together with the learning period since with longer learning periods we can obtain more precise parameter values. When the learning period is set to 600 s, the tracking success ratio is at the upper limit. In addition, the tracking success ratio is almost at the upper limit when the learning period is set to 300 s. Therefore, the learning period can be set to a value between these values.

## 5. Conclusions and Future Work

In this paper, first we presented a model of pedestrian mobility in a microcell on the basis of observation of actual pedestrian trajectories. We showed that pedestrians move along approximately straight lines and that pedestrian velocities follow a normal distribution. Based on these results, we proposed a novel method for pedestrian tracking in a microcell. In the proposed method, we extend Bayesian estimation to account for time-series information in order to estimate the correspondence between pedestrian arrival and departure events. Through simulations, we evaluated the basic performance of the proposed method in a variety of situations and demonstrated that the tracking success ratio of the proposed method is improved by 35.8% compared to a combinatorial optimization-based tracking method.

In this paper, we focused on pedestrian tracking in a microcell. In future work, we plan to develop an inter-microcell pedestrian tracking method by using results obtained in this paper. We also plan to evaluate the proposed method using actual pedestrian trajectories under different environments in terms of location, season, and time in order to investigate the feasibility of the method in real-world environments.

## Figures and Tables

**Table 1:** Parameters in the observed environment.

Parameter	Value
Cell arrival rate λ	0.208 pedestrian/s
Average velocity $\overset{-}{v}$	1.35 m/s
Variance of velocity σ^2^	5.68 × 10^−2^ m^2^/s^2^
Gate transition probability *𝒫*_transit_	Figure 7 (*n* = 80)
Gate arrival probability *𝒫*_arr_	Figure 8 (*n* = 80)

## Appendix

Theorem A.1 Assume that pedestrian velocity follows a normal distribution with a mean value $\bar{v}$ and variance *σ* ^2^. The probability density function of the distribution of pedestrian transit time *τ* necessary for a pedestrian to cover a distance *D* can be written as

(A.1) $$\begin{array}{c} p_{time}\left(\tau ,D\right)=\frac{D}{\tau^{2}}\frac{1}{\sqrt{2\pi }\sigma }\exp\left(-\frac{{\left(\left(D/\tau \right)-\bar{v}\right)}^{2}}{2\sigma^{2}}\right). \end{array}$$

Proof Since pedestrian velocity follows a normal distribution, the probability density function of the distribution of pedestrian velocities can be written as

(A.2) $$\begin{array}{c} p_{\text{vel}}\left(v\right)=\frac{1}{\sqrt{2\pi }\sigma }\exp\left(-\frac{{\left(v-\bar{v}\right)}^{2}}{2\sigma^{2}}\right). \end{array}$$

Let *F*_time_(*τ*, *D*) = *p*[*T* < *τ*] and *F*_vel_(*v*) = *p*[*V* < *v*] be the cumulative distribution functions of (A.1) and (A.2), respectively, where *V* and *T* are random variables. The following relationships exist among the probability density functions and the cumulative distribution functions:

(A.3) $$\begin{array}{c} p_{\text{vel}}\left(v\right)=\frac{d}{dv}F_{\text{vel}}\left(v\right),\\ p_{\text{time}}\left(\tau ,D\right)=\frac{d}{d\tau }F_{\text{time}}\left(\tau ,D\right). \end{array}$$

Here, *F*_time_(*τ*, *D*) can be written as follows:

(A.4) Ftime(τ,D)  =p[T<τ]=p[DV<τ]  =p[Dτ<V]=1−p[V<Dτ]  =1−Fvel(Dτ).

By differentiating (A.4) with respect to the transit time *τ*, we obtain

(A.5) ptime(τ,D)=ddτFtime(τ,D)=−ddτFvel(Dτ)=−pvel(Dτ)ddτ(Dτ)=Dτ2pvel(Dτ).

By substituting (A.2) into (A.5), we obtain (A.1).
